# Soft-Tissue Sarcomas—A Correlation Among Tumor Margin Infiltration, Immunological Markers, and Survival Rate

**DOI:** 10.3390/ijms26094363

**Published:** 2025-05-03

**Authors:** Bogdan Șerban, Adrian Cursaru, Sergiu Iordache, Mihai Costache, Bogdan Cretu, Adrian Dumitru, Catalin Cirstoiu

**Affiliations:** 1 Orthopedics and Traumatology, University Emergency Hospital, 050098 Bucharest, Romania; serbann.bogdan@yahoo.com (B.Ș.); sergiu.iordache92@yahoo.com (S.I.); mihaiaurelcostache@uahoo.com (M.C.); jfrbogdan@yahoo.com (B.C.); dr.adriandumitru@yahoo.com (A.D.); cirstoiu_catalin@yahoo.com (C.C.); 2 Orthopedics and Traumatology Department, Carol Davila University of Medicine and Pharmacy, 050474 Bucharest, Romania

**Keywords:** sarcoma, soft-tissue, tumor margin infiltration, survival rate

## Abstract

Early and appropriate diagnosis of soft-tissue sarcomas (STSs) is hampered by their relatively low prevalence and sometimes unusual clinical appearance. It takes a comprehensive diagnostic work-up to differentiate between different types of soft-tissue sarcomas. Determining tumor margins by preoperative imaging is important, especially in order to preserve the affected limb and improve quality of life. Misjudgment of tumor margins may increase or decrease the stage of soft-tissue sarcoma and thus influence the patient’s prognosis. The applicability of conventional MRI alone for determining the tumor margin is limited. Additional information regarding the peritumoral tissue, particularly at the cellular level, can be obtained via diffusion-weighted imaging (DWI). However, there are not many publications on employing DWI to evaluate tumor margin infiltration in soft-tissue sarcoma patients. Because the immune system plays a variety of roles during oncogenesis, it can occasionally be difficult to distinguish between tumor invasion and the presence of a reactive inflammatory infiltrate. Clarifying the predictive importance of lymphocyte infiltration in soft-tissue sarcomas was the goal of this investigation. We examined the correlations between expression of CD4, CD8, and CD34 and tumor margin infiltration observed on a DWI sequence. CD4, CD8, and CD34 marker positivity was linked to soft-tissue sarcomas that were less aggressive and did not invade the tumor margins, indicating a higher survival percentage for these individuals.

## 1. Introduction

According to WHO classification, soft-tissue sarcomas are a heterogenic group of malignancies that develop from the embryonic mesoderm and are divided into over 100 histological subtypes [1] based on their morphological appearance or presumed tissue of origin. Each of these subtypes has distinctive features that correlate to a different clinical course and treatment strategy.

About 13,590 new soft-tissue sarcomas will be diagnosed in the US in 2024, according to the American Cancer Society’s [2] estimations (7700 in men and 5890 in women). Soft-tissue sarcomas are expected to affect 2760 men and 2440 women, which will cause approximately 5200 deaths. Given that these statistics apply to adults as well as children, the treatment plan must be optimized (customized) from the very start of confirmed diagnosis.

Ideally, quantification of tumor characteristics ought to aid in determining the best therapy strategy for each patient with a soft-tissue neoplasm diagnosis. However, the main problem in this step is the heterogeneity of these types of sarcomas. Genetic and phenotypic variability exists between tumors and within the same tumor, known as intertumor and intratumor heterogeneity, respectively [3]. Nearly all musculoskeletal malignancies exhibit intratumor heterogeneity through several characteristics, including cellular metabolism, gene expression, and metastatic illness. With a 50% death rate [4], as was already said, it is critical to identify specific and suitable treatments to address a tumor’s aggressiveness at an early stage. Selecting the appropriate course of treatment might enhance 5-year survival, improve prognosis, and lessen the morbidity brought on by radiation or chemotherapy.

The incidence of sarcomas varies by country and by study date [5] and therefore cannot be stated with certainty. Similarly, the incidence of each individual histological subtype is unclear, and sarcomas are misdiagnosed in up to 30% of cases [6]. Thus, these misdiagnosed patients may not be managed according to clinical guidelines, which recommend that patient management be carried out by a dedicated multidisciplinary team. Given the diverse characteristics of the tumor and the disagreements over how to categorize its multiple subtypes, it has been difficult to estimate the prevalence of soft-tissue sarcomas.

## 2. Results

The study group’s case distribution showed that 51% of cases (N = 38) had invasion of the tumor margins, and there was a correlation between size and histological grade, meaning that large sarcomas with intermediate and high histological grade were linked to irregular, infiltrative margins and peritumoral edema.

**Figure 1:**
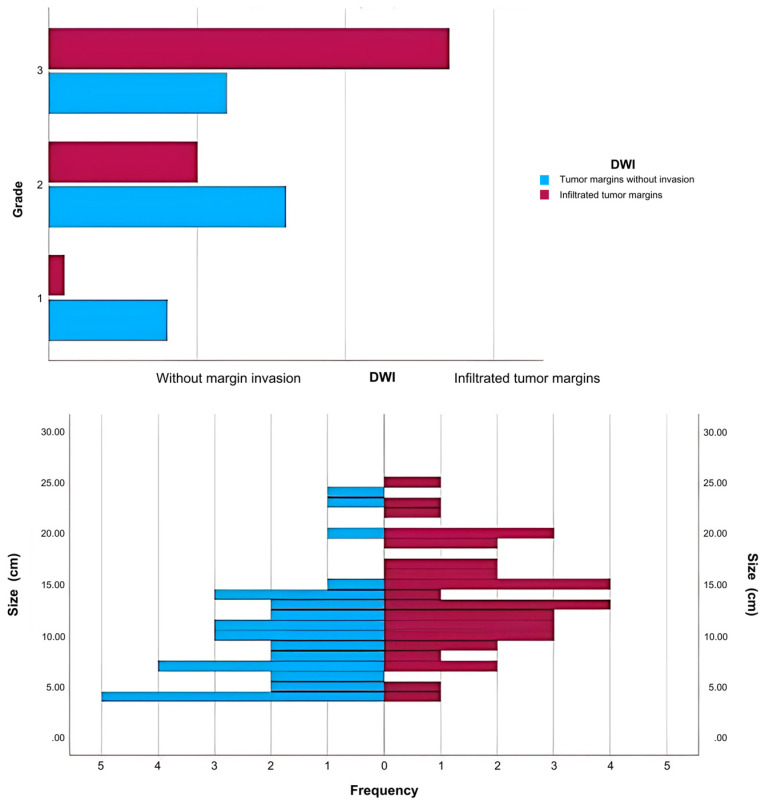
Case distribution by grade and size.

**Figure 2:**
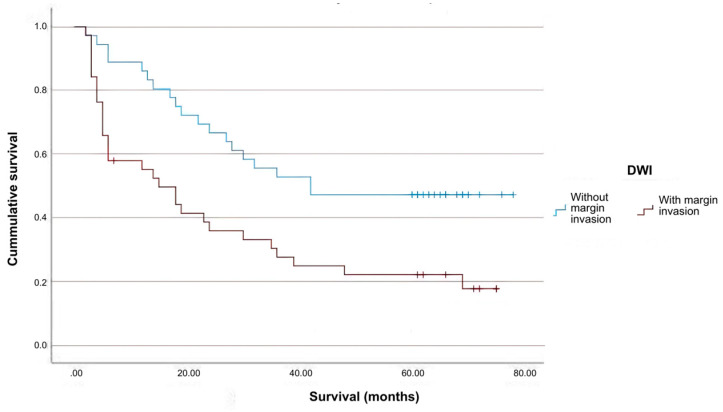
Survival analysis in DWI sequence.

Based on tumor margin invasion (as determined by MRI scan), patients were split into two groups: one with tumor margin infiltration (n = 38) and another without imaging-proven margin infiltration (n = 36). Additionally, there is a correlation between tumor margin invasion and histological grade, with tumors with intermediate and high malignancy grades exhibiting margin infiltration significantly more frequently (Figure 1). To examine the influence of tumor margin invasion on survival, a Kaplan−Meier survival analysis was conducted on this patient group (Figure 2).

**Figure 3:**
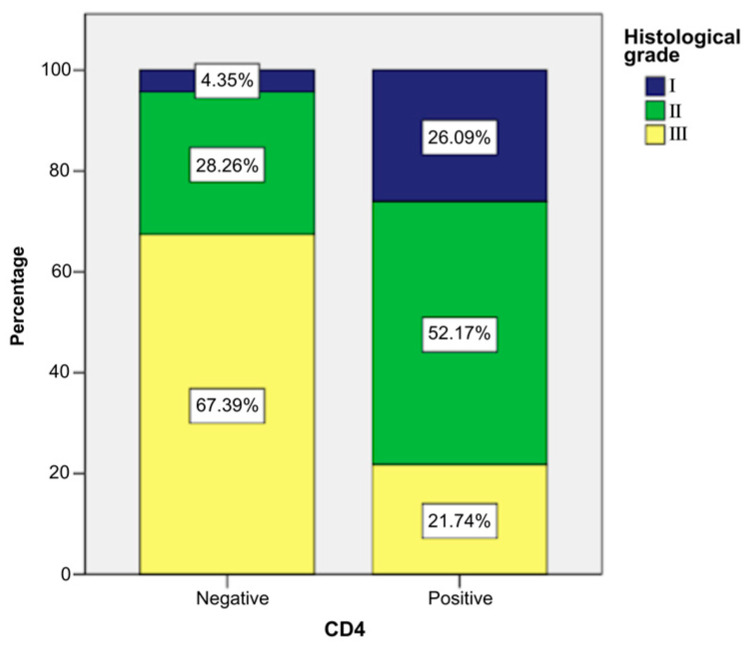
Association between the presence of CD4 and DWI.

CD4-positive patients more frequently presented low histological grade than other patients, as follows (Figure 3):

- Grade I: 26.09% of CD4-positive patients (n = 6) vs. 4.35% CD4-negative patients (n = 2).
- Grade II: 52.17% of CD4-positive patients (n = 12) vs. 28.26% CD4-negative patients (n = 13).
- Grade III: 21.74% of CD4-positive patients (n = 5) vs. 67.39% CD4-negative patients (n = 31).

The application of a chi-square test indicated that there was a statistically significant association between the presence of the marker and the grade, χ^2^ = 14.795, *p* = 0.001, and the phi and Cramer’s V coefficients (0.463) revealed that the link between the two variables was direct and moderate in intensity (*p* = 0.001). These statistical tests indicated that CD4-positive patients had lower histological grades significantly more frequently (Table S1).

**Figure 4:**
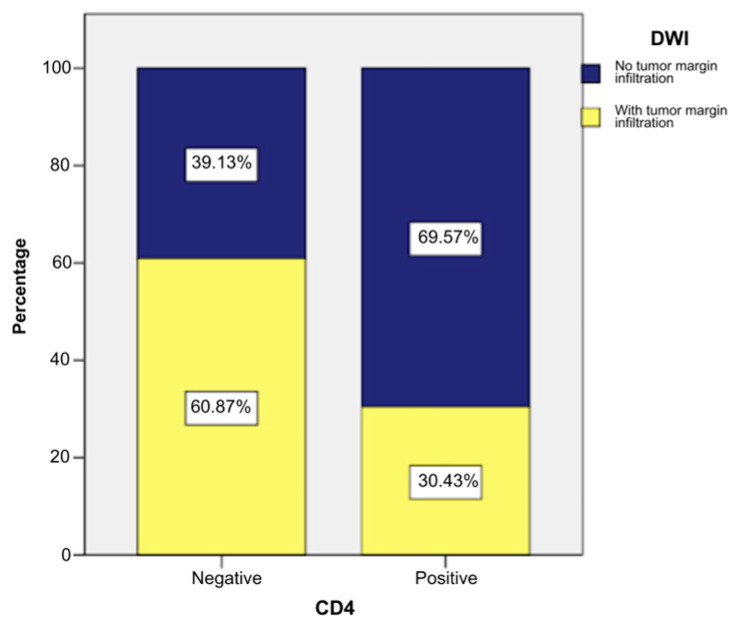
Case distribution by CD4 and DWI.

**Figure 5:**
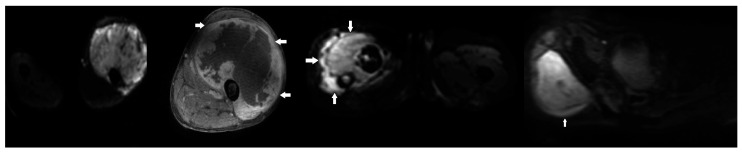
DWI scan: —tumor margin infiltration.

Tumor margin infiltration was observed less frequently in CD4-positive patients, being more common in CD4-negative cases. Thus, 30.43% of CD4-positive patients (n = 7) and 60.87% of CD4-negative patients (n = 28) had tumor margin infiltration (Figure 4 and Figure 5).

The application of a chi-square test indicated that there was a statistically significant association between the presence of the CD4 marker and DWI (tumor margin infiltration), χ^2^ = 5.682, *p* = 0.017, and the phi and Cramer’s V coefficients (−0.287) revealed that the connection between the two variables was indirect and of weak intensity (*p* = 0.017). These statistical tests indicated that CD4-positive patients had a slight tendency to present tumor margin infiltration less frequently (Table S2).

**Figure 6:**
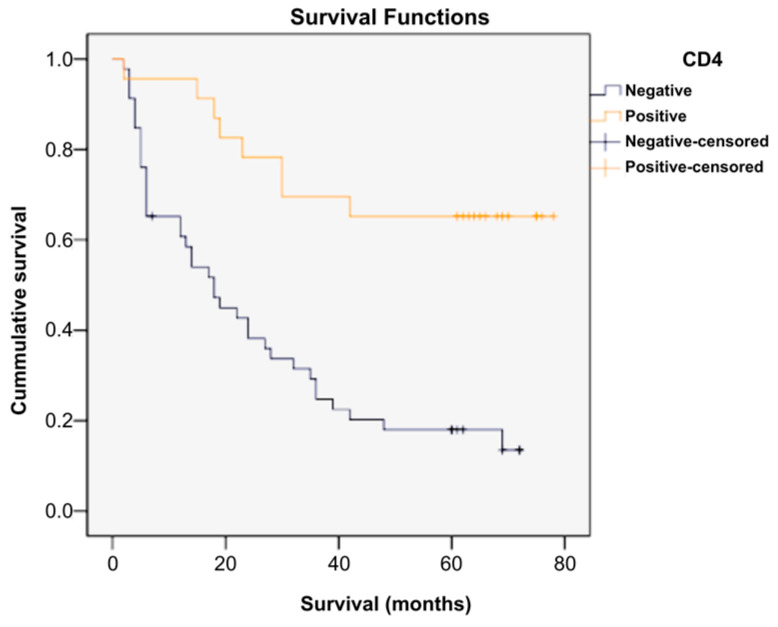
Survival rates according to CD4 presence.

Patients were divided into two different groups based on the presence of the CD4 marker: a CD4-positive group (n = 23) and a CD4-negative group (n = 46). A Kaplan–Meier survival analysis was performed to compare the effect/impact that the presence of the marker had on survival. The percentage of censored cases (survivors) in the CD4-positive group (65.20%) was different from that in the CD4-negative group (17.40%). A log-rank test was performed to determine whether there were differences between the survival distributions of the two patient groups, which were statistically significantly different, χ^2^ = 15.579, *p* ≤ 0.001. Patients with absent CD4 survived for a significantly shorter period and in a significantly lower proportion than those with positive CD4 (Figure 6, Table 1 and Table S3).

**Figure 7:**
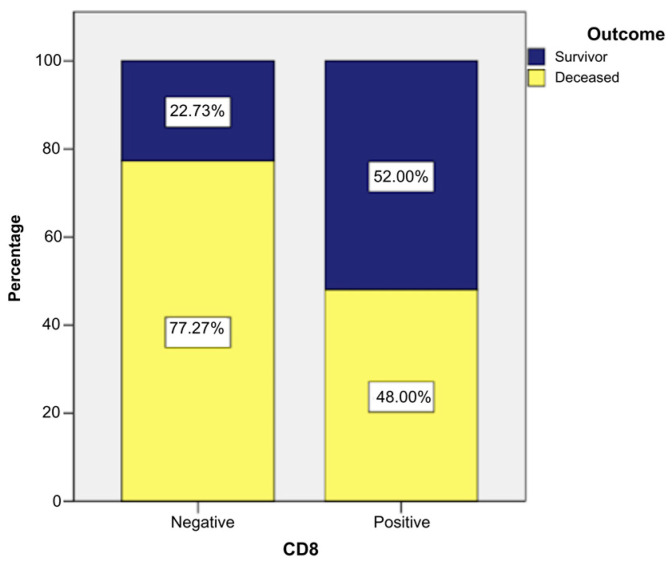
Survival rates of patients with positive/negative CD8 marker.

The presence of the CD8 marker was associated with a milder/better evolution of the sarcomas in the studied group. Cases with positive CD8 survived in 52% of cases (n = 13); in 48% of cases, they died (n = 12). Cases with negative CD8 survived in only 22.73% of cases (n = 10), while the majority, i.e., 77.27%, died (n = 34) (Figure 7).

The application of a chi-square test indicated that there was a statistically significant association between the presence of the CD8 marker and patient survival, χ2 = 6.147, *p* = 0.013, and the phi and Cramer’s V coefficients (0.298) revealed that the connection between the two variables was direct, but weak in intensity (*p* = 0.013). These statistical tests indicated that patients with positive CD8 had a significantly higher survival rate (Table S4 and Table S5).

**Figure 8:**
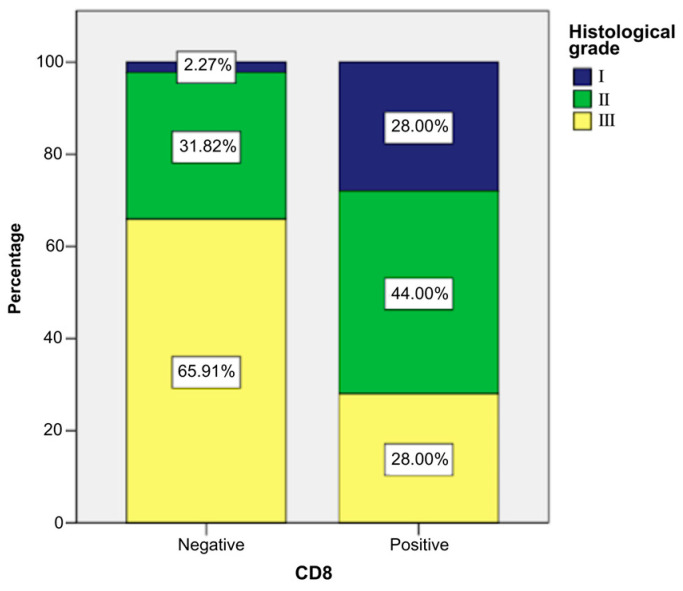
Case distribution according to CD8 and histological grade.

**Figure 9:**
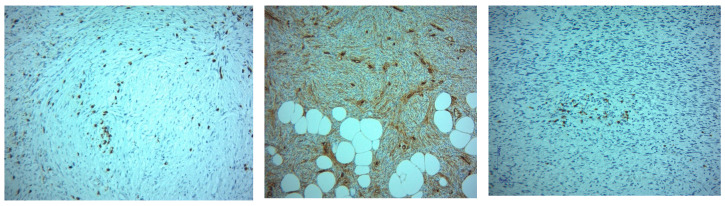
**Left** side: CD8-positive lymphocytes in small clusters or isolated, scattered between tumor cells. IHC stain with DAB chromogen, 10×; **Center**: CD34 expression reveals numerous small blood vessels, suggesting tumor neoangiogenesis at the interface with the invasion front and the adipose tissue. Isolated CD34-positive cells, probably stem-like, are also observed. IHC stain with DAB chromogen, 10×; **Right** side: Histological image of tumor margin infiltration. Note a cluster of CD4-positive T lymphocytes. IHC stain with DAB chromogen, 10×.

CD8-positive patients more frequently presented low histological grade than other patients, as follows (Figure 8 and Figure 9):

- Grade I: 28% of CD8-positive patients (n = 7) vs. 2.27% CD8-negative patients (n = 1).
- Grade II: 44% of CD8-positive patients (n = 11) vs. 31.82% CD8-negative patients (n = 14).
- Grade III: 28% of CD8-positive patients (n = 7) vs. 65.91% CD8-negative patients (n = 29).

Tumor margin infiltration was observed less frequently in CD8-positive patients, without a statistically significant association between CD8 and DWI. Thus, 40% of CD8-positive patients (n = 10) and 56.82% of CD4-negative patients (n = 25) had tumor margin infiltration (Figure 5).

Patients were divided into two different groups based on the presence of the CD8 marker: a CD8-positive group (n = 25) and a CD8-negative group (n = 44). A Kaplan–Meier survival analysis was performed to compare the effect/impact that the presence of the marker has on survival.

The percentage of censored cases (survivors) in the CD8-positive group (52%) was different from that in the CD8-negative group (22.70%). CD8-positive patients had a median time to death of 69 months, which was longer than that of CD8-negative patients, who had a median time to death of 18 months.

A log-rank test was performed to determine whether there were differences between the survival distributions of the two patient groups, which were statistically significantly different, χ^2^ = 8.272, *p* = 0.004.

**Figure 10:**
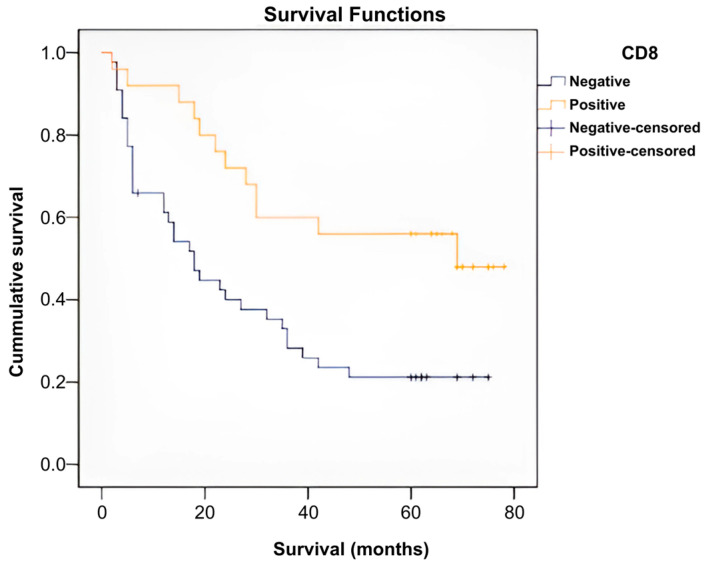
Survival curves according to the presence of CD8.

Patients with absent CD8 survived for a significantly shorter period and in a significantly lower proportion than those with positive CD8 (Figure 10, Table 2 and Table S6).

**Figure 11:**
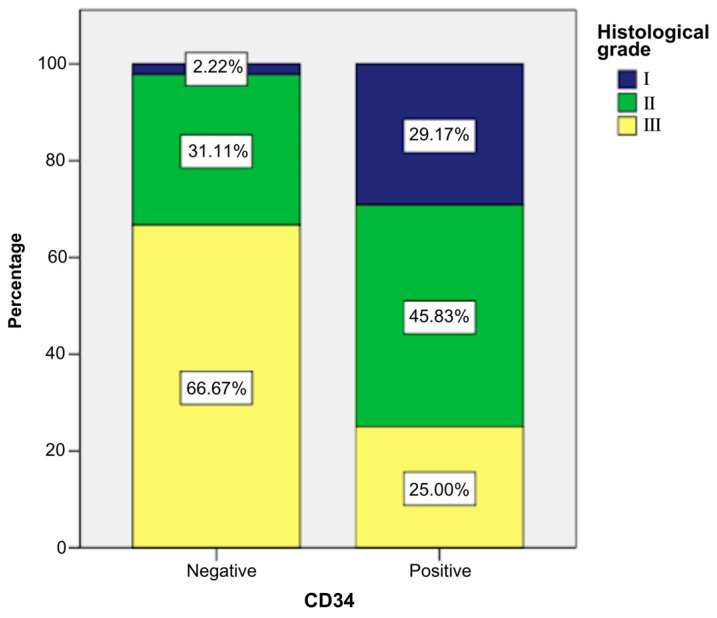
Association between patients with CD34 marker and histological grade.

CD34-positive patients more frequently presented low histological grade than other patients, as follows (Figure 11):

- Grade I: 29.17% of CD34-positive patients (n = 7) vs. 2.22% CD34-negative patients (n = 1).
- Grade II: 45.83% of CD34-positive patients (n = 11) vs. 31.11% CD34-negative patients (n = 14).
- Grade III: 25% of CD34-positive patients (n = 6) vs. 66.67% CD34-negative patients (n = 30).

The application of a chi-square test indicated that there was a statistically significant association between the presence of the marker and grade, χ^2^ = 15.946, *p* ≤ 0.001, and the phi and Cramer’s V coefficients (0.481) reveal that the link between the two variables was direct and moderate in intensity (*p* ≤ 0.001). These statistical tests indicated that CD34-positive patients had lower histological grades significantly more frequently (Table S7).

**Figure 12:**
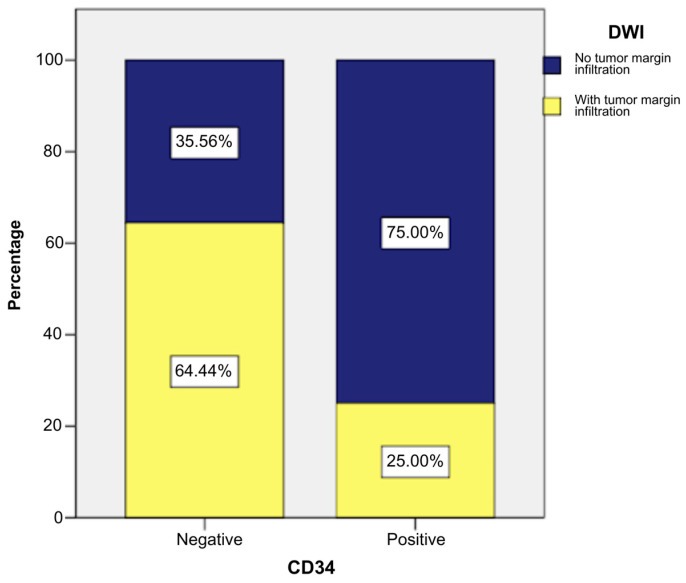
Association between the presence of CD34 marker and DWI.

Tumor margin infiltration was observed less frequently in CD34-positive patients, being more common in CD34-negative cases. Thus, 25% of CD34-positive patients (n = 6) and 64.44% of CD4-negative patients (n = 29) had tumor margin infiltration (Figure 12).

The application of a chi-square test indicated that there was a statistically significant association between the presence of the CD34 marker and DWI (tumor margin infiltration), χ^2^ = 9.743, *p* = 0.002, and the phi and Cramer’s V coefficients (−0.376) revealed that the connection between the two variables was indirect and of moderate intensity (*p* = 0.002). These statistical tests indicated that CD34-positive patients had a moderate tendency to present tumor margin infiltration less often or not to present tumor margin infiltration (Table S8).

Patients were divided into two different groups according to the presence of the CD34 marker: a CD34-positive group (n = 24) and a CD34-negative group (n = 45). A Kaplan–Meier survival analysis was performed to compare the effect/impact that the presence of the marker has on survival. The percentage of censored cases (survivors) in the CD34-positive group (65.50%) was different from that in the CD34-negative group (17.80%).

**Figure 13:**
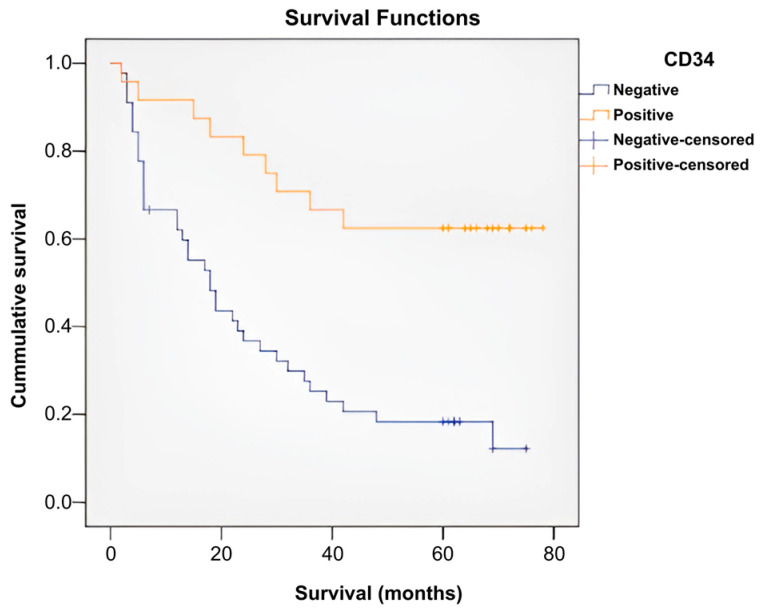
CD34 survival curves.

A log-rank test was performed to determine whether there were differences between the survival distributions of the two patient groups, which were statistically significantly different, χ^2^ = 14.719, *p* ≤ 0.001. Patients with absent CD34 survived for a significantly shorter period and in a significantly lower proportion than those with positive CD4 (Figure 13) (Table 3 and Table S9).

The presence of the CD34 marker was associated with a milder/better evolution of the sarcomas in the studied group. Cases with positive CD34 survived in 62.50% of cases (n = 15), compared with 37.50% of cases who died (n = 9). Cases with negative CD34 survived in only 17.78% of cases (n = 8), while the majority, i.e., 82.22%, died (n = 37). The application of a chi-square test indicated that there was a statistically significant association between the presence of the CD34 marker and patient survival, χ^2^ = 14.088, *p* ≤ 0.001, and the phi and Cramer’s V coefficients (0.452) revealed that the connection between the two variables was direct and moderate in intensity (*p* ≤ 0.001). These statistical tests indicated that patients with positive CD34 had a significantly higher survival rate (Table S10).

## 3. Discussion

According to the guidelines developed by the American College of Radiology, MRI is the most appropriate imaging technique for the detection and evaluation of soft-tissue sarcomas [7,8].

The applicability of conventional MRI alone for tumor margin determination is limited. Advanced imaging sequences can improve standard MRI protocols, including dynamic contrast-enhanced functional MRI (DCE) and diffusion-weighted imaging (DWI). These tools increase sensitivity and specificity, especially for the detection of local recurrence [9].

Diffusion-weighted imaging (DWI) [10] can reveal additional information about the peritumoral tissue, especially at the cellular level. There have been few reports on the assessment of tumor margin infiltration in cases of soft-tissue sarcoma using DWI.

Surgical excision is the main treatment in the case of soft-tissue sarcomas. Thus, determining the edges of the tumor through preoperative imaging studies is important, especially in order to preserve the affected limb, with the aim of improving the quality of life. Assessment of tumor extension (i.e., differentiation between peritumoral edema and peritumoral infiltration) is essential for surgical planning. Misjudgment of tumor margins can increase or decrease the stage of soft-tissue sarcoma and thus influence the patient’s prognosis [11,12].

## 4. Materials and Methods

The observational cohort analytical study was conducted over seven years (2016–2023) within the Orthopedics–Traumatology department of the Bucharest University Emergency Hospital. In accordance with the Declaration of Helsinki and approved by the ethics board, each participant received an informed consent form, and the study complied with international norms regarding the ethics and deontology of scientific research. Patient information, including age, sex, body mass index, place of birth, personal pathological history, and hereditary–collateral history, was used in the statistical analysis. Data on the extent of tumor formation, vascular or bone invasion, lymph node involvement, and the presence or absence of distant metastasis were all provided by imaging techniques (MRI, CT, Angiography, PET-CT, SPECT-CT). The study of histopathological and immunohistochemical data provided the basis for the definitive diagnosis.

The main objective of this analysis was the identification and correlation between tumor margin infiltration on MRI and the presence/absence of CD4, CD8, and CD34 lymphocyte expression in patients diagnosed with primary/recurrent soft-tissue sarcomas located in the extremities. CD4 and CD8 T lymphocytes are primarily responsible for controlling tumor development [9,13,14].

The applicability of conventional MR imaging alone for determining the tumor margin is limited.

Additional information regarding the peritumoral tissue, particularly at the cellular level, can be obtained via diffusion-weighted imaging (DWI) [11,12].

Tumor margins that were irregular or poorly delimited in the DWI sequence were categorized as infiltrated in the research, whereas well-defined, confined margins were classed as non-infiltrated. In order to ascertain the extent of extension of the examined tumor development, the peritumoral signal intensity was also assessed after the injection of the contrast agent.

## 5. Conclusions

The positivity of CD4, CD8, and CD34 markers was associated with a low degree of aggressiveness of soft-tissue sarcomas, without invasion of the tumor margins, thus illustrating a better survival rate in these patients.

The statistical analysis conducted in our study demonstrated a significantly reduced 5-year overall survival rate in the patient cohort exhibiting evidence of tumor margin infiltration.

The study highlights the importance of tumor margin infiltration and immunological markers in predicting the aggressiveness and survival outcomes of soft-tissue sarcomas. The findings suggest that assessing these factors can help in treatment strategies for better patient outcomes.

## Figures and Tables

**Table 1:** Indicators of central tendency for CD4-positive patient survival.

Means and Medians for Survival Time								
CD4	Mean ^a^				Median			
	Estimate	Std. Error	95% Confidence Interval		Estimate	Std. Error	95% Confidence Interval	
			Lower Bound	Upper Bound			Lower Bound	Upper Bound
Negative	26.325	3.593	19.283	33.366	18.000	3.302	11.527	24.473
Positive	58.652	5.693	47.494	69.811	.	.	.	.
Overall	37.738	3.660	30.565	44.911	28.000	5.125	17.956	38.044

^a^. Estimation was limited to the largest survival time if censored.

**Table 2:** Indicators of central tendency for CD8-positive patient survival.

Means and Medians for Survival Time								
CD8	Mean ^a^				Median			
	Estimate	Std. Error	95% Confidence Interval		Estimate	Std. Error	95% Confidence Interval	
			Lower Bound	Upper Bound			Lower Bound	Upper Bound
Negativ	28.709	4.070	20.732	36.687	18.000	3.227	11.674	24.326
Positive	52.360	5.724	41.141	63.579	69.000	.	.	.
Overall	37.738	3.660	30.565	44.911	28.000	5.125	17.956	38.044

^a^. Estimation is limited to the largest survival time if it is censored.

**Table 3:** Testing for differences between survival distributions of patient groups.

Means and Medians for Survival Time								
CD34	Mean ^a^				Median			
	Estimate	Std. Error	95% Confidence Interval		Estimate	Std. Error	95% Confidence Interval	
			Lower Bound	Upper Bound			Lower Bound	Upper Bound
Negativ	26.752	3.742	19.418	34.086	18.000	3.270	11.591	24.409
Pozitiv	57.083	5.736	45.840	68.327	.	.	.	.
Overall	37.738	3.660	30.565	44.911	28.000	5.125	17.956	38.044

^a^. Estimation is limited to the largest survival time if it is censored.

## Data Availability

The data presented in this study are available on request from the corresponding author due to ethical reasons.

## Supplementary Materials

The following supporting information can be downloaded at: https://www.mdpi.com/article/10.3390/ijms26094363/s1.

## Abbreviations

SPECT-CT	single photon emission tomography
STS	soft-tissue sarcomas
DWI	diffusion-weighted imaging
MRI	magnetic resonance imaging
CT	computer-tomography
IHC	Immunohistochemistry
