# Supplementary material for: Soft-Tissue Sarcomas—A Correlation Among Tumor Margin Infiltration, Immunological Markers, and Survival Rate
Source: Int J Mol Sci. 2025 May 3;26(9):4363. doi: 10.3390/ijms26094363 (PMC12072755; doi:10.3390/ijms26094363)
Supplement: Supplementary file 1 [file ijms-26-04363-s001.zip › ijms-3534636-supplementary.pdf]

Table S1. Testing the association between the presence of CD4 and histological grade.

Chi-square test			
	**Value**	**df**	Asymp. Sig. (2-sided)
Pearson Chi square	14.795[a]	2	.001
Likelihood Ratio	15.212	2	.000
Linear-by-Linear Association	14.509	1	.000
N of Valid Cases	69		
a. 1 cells (16.7%) have expected count less than 5. The minimum expected count is 2.67.			

Symmetric Measures			
		Value	Approx. Sig.
Nominal by Nominal	Phi	.463	.001
	Cramer's V	.463	.001
N of Valid Cases		69	

Table S2. Association between CD4 and DWI.

Chi-square test					
	**Value**	**df**	**Asymp. Sig. (2-sided)**	**Exact Sig. (2-sided)**	Exact Sig. (1-sided)
Pearson Chi-Square	5.682[a]	1	.017		
Continuity Correction[b]	4.530	1	.033		
Likelihood Ratio	5.794	1	.016		
Fisher's Exact Test				.022	.016
Linear-by-Linear Association	5.600	1	.018		
N of Valid Cases	69				
a. 0 cells (0.0%) have expected count less than 5. The minimum expected count is 11.33.					
b. Computed only for a 2x2 table					

Symmetric Measures			
		**Value**	**Approx. Sig.**
Nominal by Nominal	Phi	-.287	.017
	Cramer's V	.287	.017
N of Valid Cases		69	

Table S3. Testing for differences between survival distributions of patient groups.

Overall Comparisons			
	**Chi-Square**	**df**	Sig.
Log Rank (Mantel-Cox)	15.579	1	.000
Breslow (Generalized Wilcoxon)	13.660	1	.000
Tarone-Ware	14.740	1	.000
Test of equality of survival distributions for the different levels of CD4.			

Table S4. Testing the association between CD8 and death/survival.

Chi-Square Tests					
	**Value**	**df**	**Asymp. Sig. (2-sided)**	**Exact Sig. (2-sided)**	Exact Sig. (1-sided)
Pearson Chi-Square	6.147[a]	1	.013		
Continuity Correction[b]	4.901	1	.027		
Likelihood Ratio	6.057	1	.014		
Fisher's Exact Test				.018	.014
Linear-by-Linear Association	6.058	1	.014		
N of Valid Cases	69				
a. 0 cells (0.0%) have expected count less than 5. The minimum expected count is 8.33.					
b. Computed only for a 2x2 table					

Table S5. Testing the association between CD8 presence and histological grade.

Chi-square test			
	**Value**	**df**	Asymp. Sig. (2-sided)
Pearson Chi-Square	14.145[a]	2	.001
Likelihood Ratio	14.562	2	.001
Linear-by-Linear Association	13.451	1	.000
N of Valid Cases	69		
a. 1 cells (16.7%) have expected count less than 5. The minimum expected count is 2.90.			

Symmetric Measures			
		**Value**	**Approx. Sig.**
Nominal by Nominal	Phi	.453	.001
	Cramer's V	.453	.001
N of Valid Cases		69	

Table S6. Testing for differences between survival distributions of patient groups.

Overall Comparisons			
	**Chi-Square**	**df**	Sig.
Log Rank (Mantel-Cox)	8.272	1	.004
Breslow (Generalized Wilcoxon)	8.863	1	.003
Tarone-Ware	8.839	1	.003

Table S7. Testing the association between CD34 and histological grade.

Chi-Square Tests			
	**Value**	**df**	Asymp. Sig. (2-sided)
Pearson Chi-Square	15.946[a]	2	.000
Likelihood Ratio	16.395	2	.000
Linear-by-Linear Association	15.352	1	.000
N of Valid Cases	69		
a. 1 cells (16.7%) have expected count less than 5. The minimum expected count is 2.78.			

Table S8. Testing the association between CD34 and DWI.

Chi-Square Tests					
	**Value**	**df**	**Asymp. Sig. (2-sided)**	**Exact Sig. (2-sided)**	Exact Sig. (1-sided)
Pearson Chi-Square	9.743[a]	1	.002		
Continuity Correction[b]	8.229	1	.004		
Likelihood Ratio	10.074	1	.002		
Fisher's Exact Test				.002	.002
Linear-by-Linear Association	9.602	1	.002		
N of Valid Cases	69				
a. 0 cells (0.0%) have expected count less than 5. The minimum expected count is 11.83.					
b. Computed only for a 2x2 table					

Symmetric Measures			
		**Value**	**Approx. Sig.**
Nominal by Nominal	Phi	-.376	.002
	Cramer's V	.376	.002
N of Valid Cases		69	

Table S9. Testing for differences between survival distributions of patient groups.

Overall Comparisons			
	Chi-Square	df	Sig.
Log Rank (Mantel-Cox)	14.719	1	.000
Breslow (Generalized Wilcoxon)	12.455	1	.000
Tarone-Ware	13.674	1	.000
Test of equality of survival distributions for the different levels of CD34.			

Table S10. Testing the association between CD34 and death/survival.

Chi-Square Tests					
	**Value**	**df**	**Asymp. Sig. (2-sided)**	**Exact Sig. (2-sided)**	Exact Sig. (1-sided)
Pearson Chi-Square	14.088[a]	1	.000		
Continuity Correction[b]	12.147	1	.000		
Likelihood Ratio	13.963	1	.000		
Fisher's Exact Test				.000	.000
Linear-by-Linear Association	13.883	1	.000		
N of Valid Cases	69				
a. 0 cells (0.0%) have expected count less than 5. The minimum expected count is 8.00.					

Overall Comparisons			
	**Chi-Square**	**df**	**Sig.**
**Log Rank (Mantel-Cox)**	**14.719**	**1**	**.000**

**Breslow (Generalized Wilcoxon)**	**12.455**	**1**	**.000**
**Tarone-Ware**	**13.674**	**1**	**.000**

Test of equality of survival distributions for the different levels of CD34.
